# Danggui Buxue Decoction and Its Active Constituents Inhibit Drug-Induced Uterine Contractions via L-Type Calcium Channels and the IP_3_/Ca^2+^ Pathway

**DOI:** 10.3390/ph19030520

**Published:** 2026-03-23

**Authors:** Mingming Liu, Taiping He, Wenqiao An, Pengmei Guo, Tang Zhou, Yufei Chen, Xiaojuan Tian, Mingxu Wu, Ting Zhang, Sanyin Zhang

**Affiliations:** 1 School of Basic Medicine, Chengdu University of Traditional Chinese Medicine, Chengdu 611137, China; liumingming@stu.cdutcm.edu.cn (M.L.); hetaiping@stu.cdutcm.edu.cn (T.H.); anwenqiao2@stu.cdutcm.edu.cn (W.A.); tianxiaojuan@stu.cdutcm.edu.cn (X.T.); mingxuwtcm@163.com (M.W.); 2 Innovative Institute of Chinese Medicine and Pharmacy, Chengdu University of Traditional Chinese Medicine, Chengdu 611137, China; guopengmei@cdutcm.edu.cn (P.G.); zhoutang20210415@163.com (T.Z.); 3 School of Medical Technology, Chengdu University of Traditional Chinese Medicine, Chengdu 611137, China; chenyufei1207@163.com

**Keywords:** Danggui Buxue Decoction, uterine smooth muscle, primary dysmenorrhea, L-type calcium channels, Ca^2+^

## Abstract

**Background/Objectives**: Primary dysmenorrhea is a common gynecological disorder characterized by painful uterine contractions. Danggui Buxue Decoction (DBD) is used to treat menstrual irregularities, but its mechanism in primary dysmenorrhea remains unclear. This study investigated the efficacy of DBD against dysmenorrhea and its calcium signaling-related mechanism. **Methods**: DBD components were analyzed by UPLC–Orbitrap MS. Isolated uterine muscle strips precontracted with oxytocin (OT, 50 ng/mL) or KCl (60 mM) were used to assess the effects of DBD and its active compounds (Quercetin, Formononetin, Ononin, Ferulic acid, Senkyunolide I, Calycosin, Ligustilide, Calycosin-7-O-β-D-glucoside). Ca^2+^-dependent experiments, intracellular calcium release assays, and inhibitor treatments (Nifedipine, 2-APB) were performed to evaluate the involvement of L-type calcium channels and the IP_3_R pathway. A primary dysmenorrhea model induced by estradiol benzoate and oxytocin was used to assess the analgesic effects, histopathology, inflammatory factors, and IP_3_/Ca^2+^-related proteins and genes following DBD and Quercetin treatment. **Results**: A total of 161 compounds were identified in DBD. DBD and its eight active constituents relaxed OT (50 ng/mL) or KCl (60 mM)-induced uterine contractions, with Quercetin, Calycosin, and Ligustilide showing particularly prominent relaxant activity. These three compounds suppressed extracellular calcium influx and intracellular calcium release through the blockade of L-type calcium channels and IP_3_R. In vivo, DBD and Quercetin alleviated pain, reduced inflammation, and decreased uterine Ca^2+^ and IP_3_ levels in dysmenorrhea mice. **Conclusions**: DBD and its active component Quercetin promote uterine relaxation by lowering Ca^2+^ levels, which is achieved through suppression of L-type calcium channels and the IP_3_/Ca^2+^ pathway. This contributes to their therapeutic action against primary dysmenorrhea.

## 1. Introduction

The pathogenesis of primary dysmenorrhea is closely associated with abnormal uterine contractions [1]. Excessive contraction of uterine muscle results in transient uterine ischemia and hypoxia, thereby inducing pain symptoms [2]. Primary dysmenorrhea is a common gynecological condition involving recurrent, cramping lower abdominal pain during menstruation, despite the absence of detectable pelvic pathology [3]. Systemic symptoms such as nausea, vomiting, cold extremities, and syncope may accompany severe dysmenorrhea, with consequent impairment of patients’ quality of life and work capacity [4,5]. The global prevalence of primary dysmenorrhea was found to be 73% (95% confidence interval [CI]: 68–78%), with higher rates observed in adults (73.3%) and university students (78.4%) [6]. Severe pain affects up to 29% of affected girls with dysmenorrhea [7]. The proportion of adolescents missing school due to dysmenorrhea ranges from 7.7% to 57.8%, and 21.5% of women report disruption to their social activities [8]. Despite the significant impact of menstrual pain on women’s lives, the survey found that 93.2% of sufferers did not seek medical advice, and 82% opted for self-medication [9].

Some evidence indicates that Calcium signaling plays a critical role in regulating uterine contractility [10]. In smooth muscle cells, intracellular Ca^2+^ is mainly derived from two sources: release from the sarcoplasmic reticulum and influx through L-type calcium channels [11,12]. Oxytocin and PGF2α activate GPCRs, activating PLC to convert PIP_2_ to IP_3_ and DAG. IP_3_ binds IP_3_Rs on the sarcoplasmic reticulum, releasing Ca^2+^ from intracellular stores [13]. An increase in intracellular calcium ions enables calcium ions to bind with calmodulin (CaM) and activate myosin light chain kinase (MLCK) [14,15]. MLCK phosphorylates the myosin light chain regulator (MLC_20_), altering the conformation of the myosin head and facilitating the formation of cross-bridges with actin to generate tension that induces cellular contraction [16].

For the treatment of primary dysmenorrhea, non-steroidal anti-inflammatory drugs (NSAIDs), including aspirin and ibuprofen, represent a first-line therapeutic option. Although NSAIDs can alleviate pain in primary dysmenorrhea patients, they also cause adverse reactions affecting the gastrointestinal and nervous systems, such as nausea, vomiting, indigestion, headaches, and drowsiness [17]. Furthermore, about 18% of primary dysmenorrhea patients experience little to no pain relief from NSAIDs [18]. Traditional Chinese medicinal formulas including Danggui Sini Decoction [19], Wenjing decoction [20], and Ge-gen decoction [21] have shown appreciable efficacy in treating primary dysmenorrhea and relieving pain.

Danggui Buxue Decoction (DBD), a traditional Chinese herbal formula, contains *Astragalus membranaceus* (AR) and *Angelica sinensis* (AES) in a 5:1 ratio [22]. First recorded in Li Dongyuan’s Discerning Confusions in Internal and External Injuries, this formula serves as a classic example of a prescription that tonifies qi and promotes blood generation. DBD is commonly used to address symptoms such as fatigue-induced thirst, physical weakness and exhaustion, fever during menstruation or postpartum, and anemia [23]. DBD can enhance energy metabolism, improve hematopoietic function, and exert anti-inflammatory and antioxidant effects [24,25,26,27,28]. Moreover, it inhibits vascular smooth muscle contraction [29,30]. AES alone has been shown to be effective in treating menstrual irregularities and amenorrhea, and providing pain relief [31,32]. These findings collectively suggest DBD’s therapeutic potential for primary dysmenorrhea; however, reports on its specific application in primary dysmenorrhea treatment remain limited.

In this study, UPLC–Orbitrap MS was employed to analyze the chemical constituents of DBD. The uterine smooth muscle relaxant activity of these constituents was evaluated, and the preliminary mechanism of the most effective components was investigated using in vivo and in vitro models. Consequently, this study investigated the mechanism underlying the uterine-relaxant effect of DBD and its active component, and evaluated its efficacy in a mouse model of primary dysmenorrhea.

## 2. Results

### 2.1. UPLC–Orbitrap MS Analysis of DBD and Identification of the Main Components

**Figure 1:**
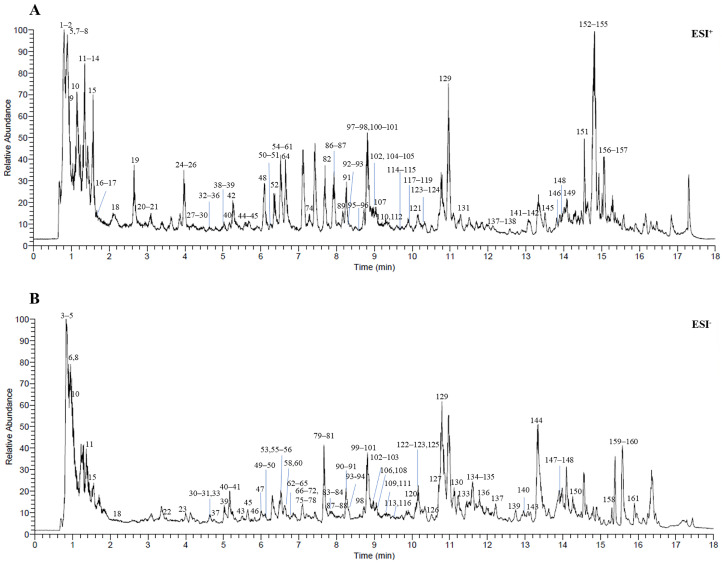
Representative UPLC–Orbitrap MS chromatograms of DBD: (**A**) ESI^+^ mode; (**B**) ESI^−^ mode.

In this study, a sensitive, reliable, and high-throughput UPLC–Orbitrap MS method was established for rapid identification of chemical constituents in DBD. Analysis of DBD extract under both positive and negative ion modes yielded the total ion chromatogram (TIC) shown in Figure 1. Based on mass spectrometry fragmentation patterns and literature data, 161 compounds were tentatively identified, comprising primarily flavonoids, phthalides, and polyphenolic compounds. Specifically, these included 52 flavonoids, 23 phthalides, 21 polyphenols, 9 triterpenoids, and 56 compounds from other structural classes (Table 1).

### 2.2. DBD, AR and AES Effectively Relaxed OT (50 ng/mL) or KCl (60 mM)-Induced Uterine Contractions

**Figure 2:**
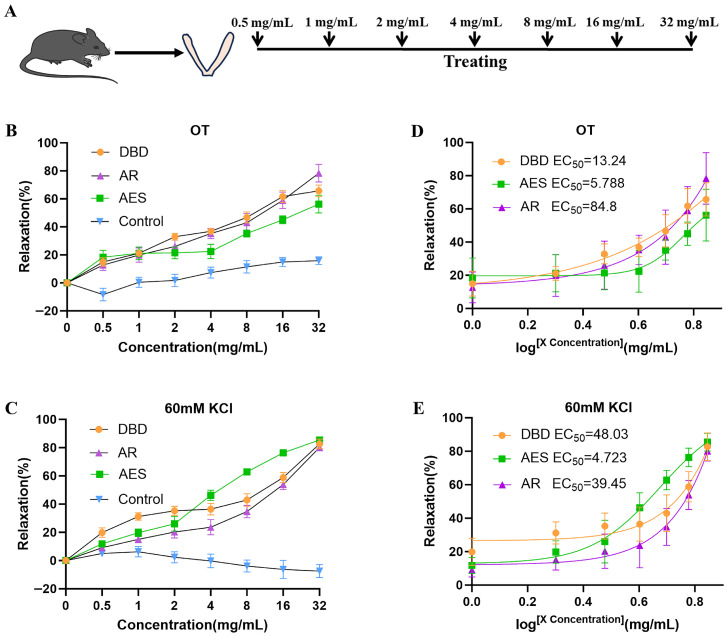
DBD, AR and AES effectively relaxed OT (50 ng/mL) or KCl (60 mM)-induced uterine contractions: (**A**) Workflow of the in vitro uterine contraction assay. (**B**,**D**) DBD, AR and AES on the uterine relaxation rate and EC_50_ fitting curves under OT (50 ng/mL) precontracted conditions. (**C**,**E**) DBD, AR and AES on the uterine relaxation rate and EC_50_ fitting curves under KCl (60 mM) precontracted conditions. Data are presented as mean ± SEM (n = 6). n = number of uterine muscle strips (one per mouse).

To investigate whether DBD could inhibit uterine contractions, we established an isolated uterine muscle strip contraction model induced by OT (50 ng/mL) or KCl (60 mM) (Figure 2A). DBD, AR, and AES each exhibited concentration-dependent relaxation of uterine muscle strips precontracted by OT (50 ng/mL) or KCl (60 mM), compared with the control group (Figure 2B,C). The EC_50_ values of DBD, AR, and AES for inhibiting OT (50 ng/mL)-induced uterine contractions were 13.24, 84.8, and 5.788 mg/mL, respectively (Figure 2D). Similarly, their EC_50_ values against KCl (60 mM)-induced contractions were 48.03, 39.45, and 4.723 mg/mL, respectively (Figure 2E). This verifies that DBD, AES and AR have the potential to inhibit uterine contraction.

### 2.3. The Eight Active Constituents of DBD-Relaxed OT (50 ng/mL) or KCl (60 mM)-Induced Uterine Contraction

**Figure 3:**
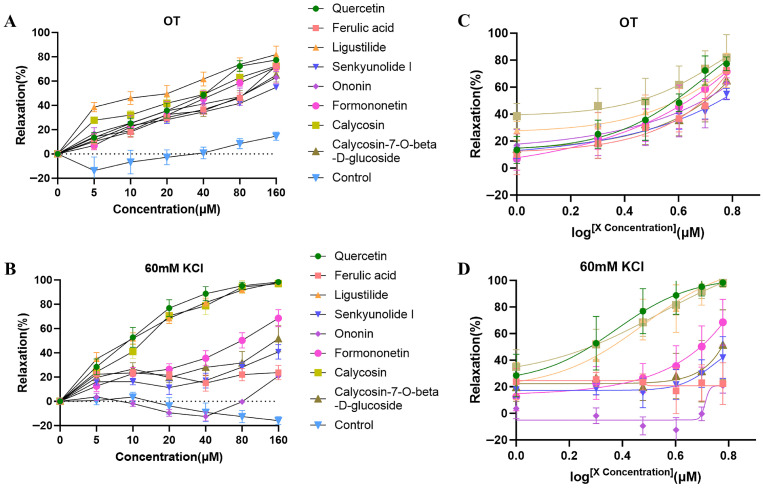
The eight active constituents of DBD-relaxed OT (50 ng/mL) or KCl (60 mM)-induced uterine contraction: (**A**,**C**) Effects of the active constituents of DBD on uterine relaxation rate and EC_50_ fitting curves under OT (50 ng/mL) precontracted conditions. (**B**,**D**) Effects of the active constituents of DBD on uterine relaxation rate and EC_50_ fitting curves under KCl (60 mM) precontracted conditions. Data are presented as mean ± SEM (n = 6). n = number of uterine muscle strips (one per mouse).

As shown in Figure 3A, all eight components relaxed the uterine contractions induced by OT (50 ng/mL) at concentrations of 5, 10, 20, 40, 80, and 160 μM. In contrast, when uterine contractions were induced by KCl (60 mM), all components except Ononin (which showed relaxation only at 80 and 160 μM) produced relaxing effects (Figure 3B). The uterine smooth muscle relaxant activity varied among the eight compounds, with Quercetin, Calycosin, and Ligustilide displaying the highest potency. Their EC_50_ values were 35.49, 59.82, and 47.23 μM for OT (50 ng/mL)-induced contractions (Figure 3C, Table 2); and 8.911, 12.10, and 11.15 μM for KCl (60 mM)-induced contractions (Figure 3D, Table 2). These results suggest that the relaxant effect of DBD on female mice uterine contractions may be closely related to three active components.

### 2.4. DBD, Quercetin, Calycosin and Ligustilide Reduce Ca^2+^ Levels

**Figure 4:**
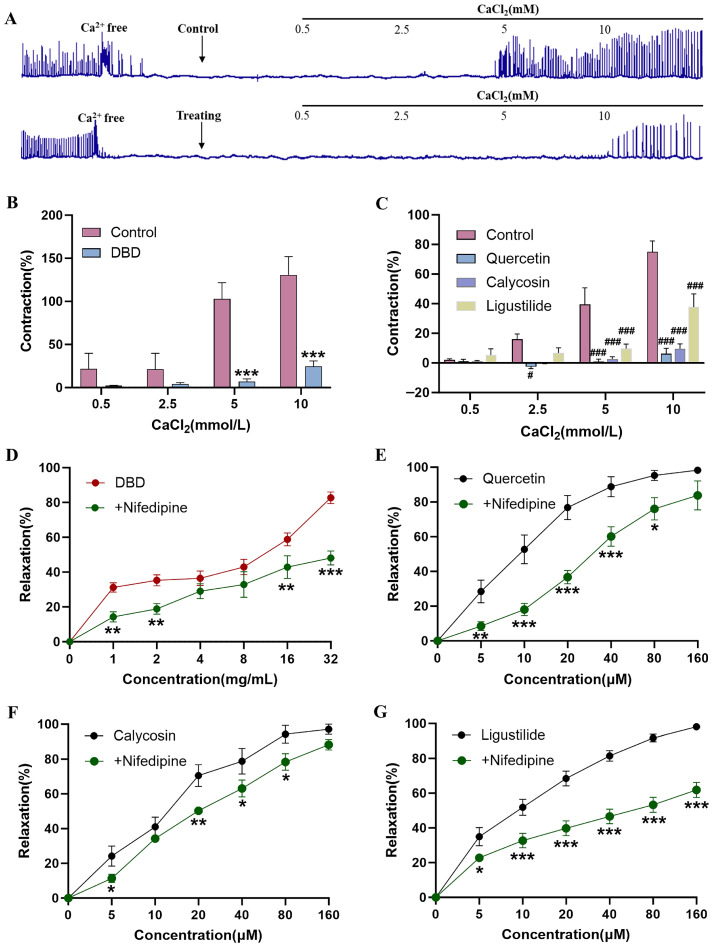
Effects of DBD, Quercetin, Calycosin and Ligustilide on Ca^2+^: (**A**) Representative graphs of Ca^2+^-dependent contraction responses to DBD, Quercetin, Calycosin and Ligustilide. (**B**,**C**) Inhibitive effect of DBD, Quercetin, Calycosin and Ligustilide on the mean peak amplitude. (**D**–**G**) Nifedipine attenuated the uterine-relaxant effects of DBD, Quercetin, Calycosin and Ligustilide. Compared with the control group, DBD showed * *p* < 0.05, ** *p* < 0.01, *** *p* < 0.001; Quercetin, Calycosin and Ligustilide showed ^#^ *p* < 0.05, ^###^ *p* < 0.001. Data are presented as mean ± SEM (n = 6). n = number of uterine muscle strips (one per mouse).

Calcium signaling plays a critical role in regulating uterine contractility. An exogenous Ca^2+^ supplementation experiment using CaCl_2_ was conducted to determine whether Ca^2+^ participate in the uterine-relaxing effects of DBD, Quercetin, Calycosin and Ligustilide. The addition of external calcium (0.5–10 mM) restored spontaneous contractions in uterine muscle strips (Figure 4A). However, incubation with DBD, Quercetin, Calycosin or Ligustilide suppressed these restored contractions in a concentration-dependent manner (Figure 4B,C). These findings suggest that the uterine-relaxant effects of DBD, Quercetin, Calycosin and Ligustilide are Ca^2+^ related.

To investigate the role of Ca^2+^, cell membranes were depolarized using KCl (60 mM) to permit extracellular calcium influx through L-type calcium channels. Pre-incubation with Nifedipine attenuated the uterine-relaxant effects of DBD, Quercetin, Calycosin and Ligustilide (Figure 4D–G). Among these, Ligustilide was the most strongly inhibited by Nifedipine. These findings demonstrate that DBD, Quercetin, Calycosin and Ligustilide induce uterine relaxation primarily by inhibiting L-type calcium channels.

**Figure 5:**
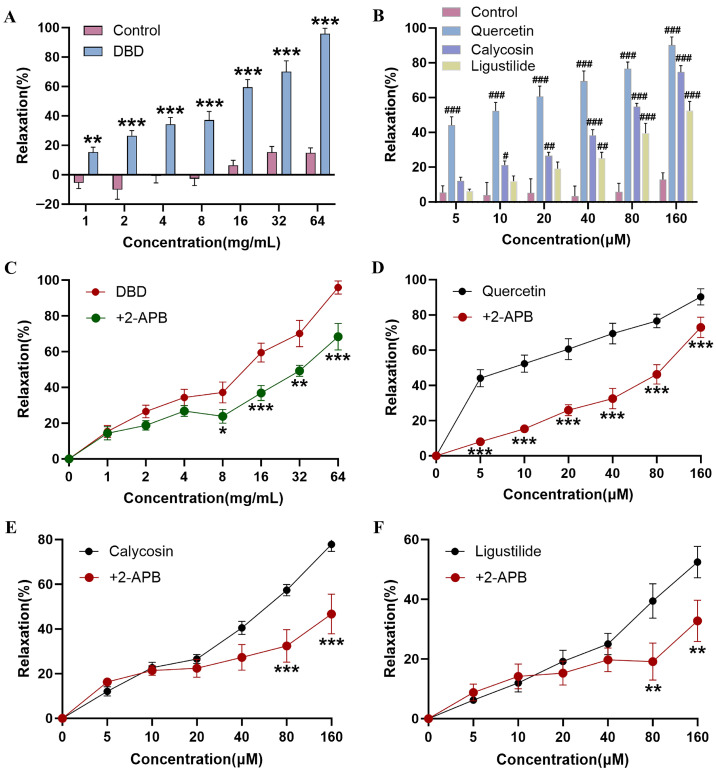
DBD, Quercetin, Calycosin and Ligustilide inhibit intracellular calcium release: (**A**,**B**) DBD, Quercetin, Calycosin and Ligustilide inhibit intracellular calcium release. (**C**–**F**) 2-APB attenuated the uterine-relaxant effects of DBD, Quercetin, Calycosin and Ligustilide. Compared with the control group, DBD showed * *p* < 0.05, ** *p* < 0.01, *** *p* < 0.001; Quercetin, Calycosin and Ligustilide showed ^#^ *p* < 0.05, ^##^ *p* < 0.01, ^###^ *p* < 0.001. Data are presented as mean ± SEM (n = 6). n = number of uterine muscle strips (one per mouse).

In the intracellular calcium release experiment, DBD, Quercetin, Calycosin and Ligustilide suppressed OT (50 ng/mL)-elicited contractions in a concentration-dependent fashion (Figure 5A,B). Among the compounds tested, Quercetin exhibited the strongest uterine-relaxant effect. To explore the mechanisms, the IP_3_R antagonist 2-APB was used to preliminarily validate whether DBD, Quercetin, Calycosin and Ligustilide inhibit intracellular calcium release via this pathway. Pre-incubation with 2-APB attenuated the uterine-relaxant effects of DBD, Quercetin, Calycosin and Ligustilide (Figure 5C–F). Among these, Quercetin exhibited the strongest uterine-relaxant effect, while Calycosin and Ligustilide demonstrated significant uterine-relaxant activity at concentrations ranging from 80 to 160 μM. These results suggest that DBD, Quercetin, Calycosin and Ligustilide exert uterine relaxation primarily via IP_3_R inhibition, a key event in suppressing the IP_3_/Ca^2+^ path.

### 2.5. DBD Alleviates Pain and Uterine Inflammation in Dysmenorrhea Mice

**Figure 6:**
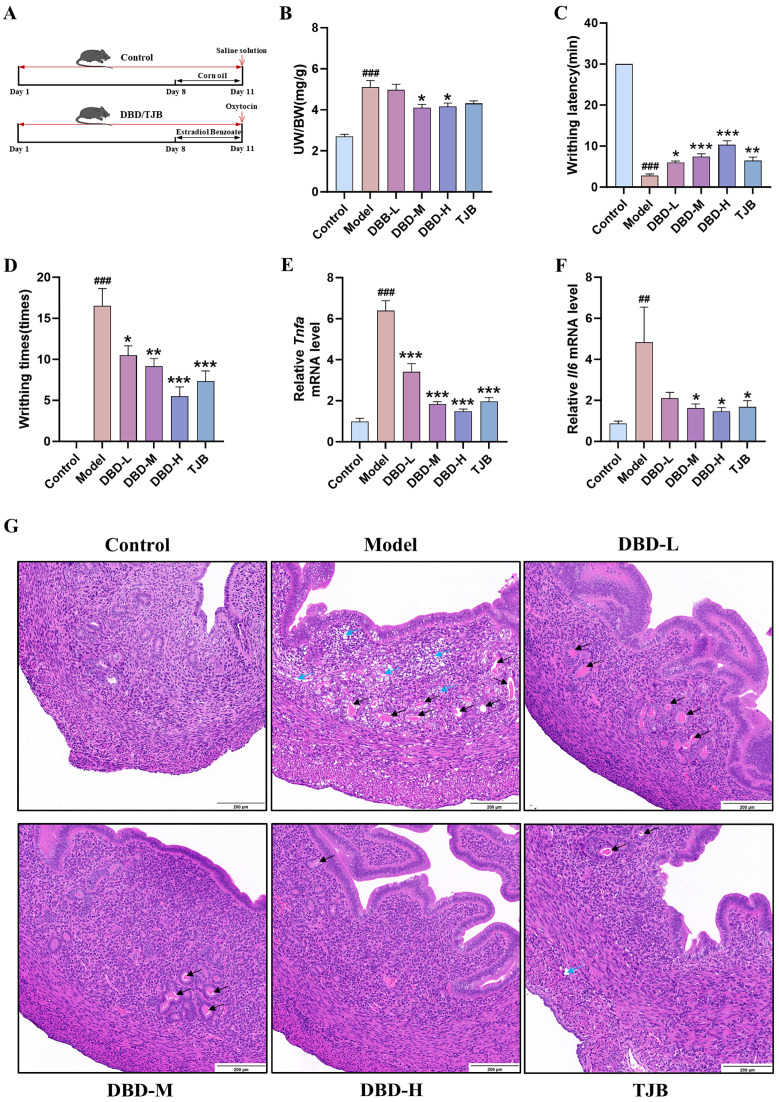
Effect of BDB on primary dysmenorrhea female mice: (**A**) Flowchart of the establishment and treatment protocol for a primary dysmenorrhea mouse model. The uterine organ index (**B**), writhing latency (**C**), and writhing times (**D**) in primary dysmenorrhea model mice. (**E**,**F**) Expression of *Tnfa* and *Il6* mRNA in uterine tissue. (**G**) Uterine HE-stained sections: black arrows indicate glandular hyperplasia and increased secretion within the intrinsic layer; blue arrows indicate interstitial edema. ^##^ *p* < 0.01, ^###^ *p* < 0.001 vs. control group, * *p* < 0.05, ** *p* < 0.01, *** *p* < 0.001 vs. model group. Data are presented as mean ± SEM (n = 6).

To assess the therapeutic effects of DBD and Quercetin against dysmenorrhea, an OT-induced dysmenorrhea model was employed in female mice (Figure 6A). Successful establishment of the dysmenorrhea model was confirmed by significant changes in uterine organ index, writhing latency, and writhing times in model female mice compared with the control group (Figures S2 and S3). After DBD pretreatment, DBD administration reduced the uterine organ index, prolonged the writhing latency, and decreased the writhing frequency (maximum inhibition rates of 70.8%, 79.2%, 87.5%, and 83.3%) (Figure 6B–D). In female mice uterine tissue, mRNA levels of *Tnfa* and *Il6* were notably increased in the model group relative to controls. Treatment with either DBD or TJB significantly reduced the expression of these pro-inflammatory genes (Figure 6E,F). Compared with the control group, the model group exhibited structural disorganization, increased glandular secretion, and marked inflammatory infiltration with edema; conversely, DBD treatment reduced inflammatory cell influx and alleviated tissue edema. (Figure 6G). These results demonstrate that DBD alleviates pain symptoms, inflammation, and pathological changes in dysmenorrhea female mice.

### 2.6. DBD and Quercetin Alleviate Dysmenorrhea by Reducing Ca^2+^ Levels

**Figure 7:**
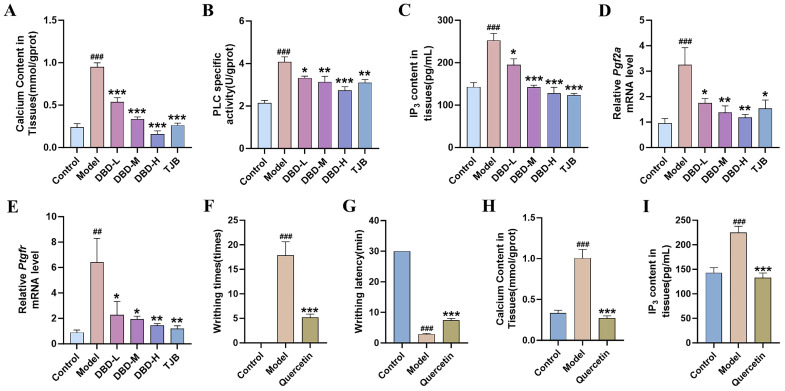
DBD and Quercetin alleviate dysmenorrhea by reducing Ca^2+^ levels: Changes in Ca^2+^ (**A**), PLC (**B**) and IP3 (**C**) in uterine tissue homogenates from primary dysmenorrhea female mice. (**D**,**E**) Expression of *Pgf2a* and *Ptgfr* mRNA in uterine tissue. (**F**) Writhing times; (**G**) Writhing latency; Ca^2+^ levels (**H**); and IP_3_ levels (**I**) in uterine tissue homogenates. ^##^ *p* < 0.01, ^###^ *p* < 0.001 vs. control group, * *p* < 0.05, ** *p* < 0.01, *** *p* < 0.001 vs. model group. Data are presented as mean ± SEM (n = 6).

To determine whether Ca^2+^ signaling mediates the anti-dysmenorrhea effect of DBD, we assessed intracellular Ca^2+^ concentrations in uterine tissue. Dysmenorrhea female mice exhibited elevated Ca^2+^ levels compared with those from control mice. Conversely, these increased levels were significantly reduced by treatment with either DBD or TJB (Figure 7A). PLC and IP_3_ levels were elevated, whereas both DBD and TJB treatments effectively lowered their concentrations in uterine tissue (Figure 7B,C). RT-qPCR was performed to measure the expression of *Pgf2a* and its receptor *Ptgfr*. DBD or TJB treatment suppressed the upregulation of *Pgf2a* and *Ptgfr* mRNA in model female mice (Figure 7D,E).

Molecular docking analysis revealed favorable binding affinities for all eight active components with PTGFR (binding energies < −5.0 kcal/mol), particularly Quercetin (binding energies −8.4 kcal/mol), which exhibited the lowest binding energy (Figure S4, Table S1). Experimental results of Quercetin treatment for dysmenorrhea indicate that, compared with the model group, Quercetin reduced writhing times, prolonged the writhing latency, and decreased Ca^2+^ and IP_3_ levels (Figure 7F,G). The findings presented above suggest that DBD and Quercetin may alleviate dysmenorrhea by lowering calcium ion levels through the IP_3_/Ca^2+^ pathway.

## 3. Discussion

In the present study, we evaluated the uterine-relaxant effects of DBD and its active components, as well as their efficacy in treating primary dysmenorrhea, by establishing an in vitro uterine contraction model and an in vivo mouse model of dysmenorrhea. Research has revealed that these effects are achieved by inhibiting L-type calcium channels and the IP_3_/Ca^2+^ pathway, thereby reducing intracellular calcium ion concentrations within the uterus and consequently exerting a uterine-relaxant effect. These findings provide experimental evidence for the potential use of DBD and its active component, Quercetin, in treating primary dysmenorrhea, demonstrating that their therapeutic mechanism involves the inhibition of calcium pathways to relax uterine smooth muscle.

Currently, DBD is employed in the treatment of female menopausal syndrome due to its estrogenic effects without inducing estrogenic side effects [33]. Combination therapy with DBD and doxorubicin effectively reduces tumor cell proliferation in triple-negative breast cancer [34]. Clinical studies have demonstrated that proprietary Chinese medicines containing AES and AR, such as Wuji Baifeng Pills and Ai Fu Nuan Gong Pills [35,36,37], effectively alleviate pain symptoms in patients with dysmenorrhea without causing serious adverse reactions. According to traditional Chinese medicine theory, therapeutic principles such as tonifying qi and blood, and promoting blood circulation to resolve stasis, play important roles in the treatment of primary dysmenorrhea [38]. DBD is a classic formula for tonifying qi and generating blood, frequently used to alleviate symptoms such as fever and anemia in women during menstruation and the postpartum period [23]. Compared to other formulas for dysmenorrhea, such as Danggui Sini Decoction and Wenjing decoction, DBD offers several advantages. Its simple two-herb composition is associated with potent efficacy, absence of known toxicity, and flexibility for clinical modification. A notable advantage of DBD lies in its organ-selective estrogen-like effects, which occur without the reproductive organ proliferation typically associated with estrogen stimulation [39].

Using UPLC–Orbitrap MS, 161 compounds were identified in DBD, many of which exhibited significant biological activities, including anti-inflammatory, analgesic, and uterine smooth muscle relaxant effects. A study reported that Senkyunolide I, Senkyunolide H, Ligustilide and Z-butylidene phthalide can relax uterine smooth muscle [40]. Quercetin can relax isolated porcine uteri [41]. The highest concentrations of DBD were found in cardiac and uterine tissues, with six compounds, including Formononetin, Ononin, Senkyunolide I, Calycosin, Ligustilide, and Calycosin-7-O-β-D-glucoside, detected in the uterine tissue [42]. Pharmacokinetic investigations revealed that following DBD administration to rats, compounds including calycosin-7-O-β-D-glucoside, Ononin, and Ferulic acid were detectable in plasma [43,44]. Based on the above rationale, the following eight compounds were selected for further investigation: Quercetin, Formononetin, Ononin, Ferulic acid, Senkyunolide I, Calycosin, Ligustilide, and Calycosin-7-O-β-D-glucoside. In experiments involving OT (50 ng/mL) or KCl (60 mM)-induced contractions in isolated uterine tissue, DBD, AES, AR, and the eight compounds were observed to induce relaxation of uterine smooth muscle, albeit to varying degrees. Among these, Quercetin, Calycosin, and Ligustilide tended to exhibit more pronounced effects.

Changes in intracellular Ca^2+^ concentration in uterine smooth muscle are essential for initiating, sustaining, and modulating the intensity of contractions [45]. Previous studies have demonstrated that Quercetin and Ligustilide induce relaxation of isolated uterine muscle strips through a calcium-mediated mechanism [46,47]. The Ca^2+^-dependent experimental results showed that uterine muscle strips pre-incubated with DBD, Quercetin, Calycosin or Ligustilide did not effectively recover contractile activity, even after the addition of exogenous CaCl_2_. Therefore, calcium is involved in the uterine-relaxant effects of DBD and its active constituents. L-type calcium channels are considered the primary ion channels mediating Ca^2+^ influx into smooth muscle cells [48]. Intracellular IP_3_ binds to IP_3_R on the sarcoplasmic reticulum, leading to the release of calcium ions [49]. After pretreatment with the L-type calcium channel blocker Nifedipine or the IP_3_R inhibitor 2-APB, the uterine-relaxant effects of DBD, Quercetin, Calycosin and Ligustilide were attenuated. These findings indicate that DBD, Quercetin, Calycosin and Ligustilide lower calcium ion levels, probably by suppressing L-type calcium channels and IP_3_R, leading to uterine relaxation.

Primary dysmenorrhea is also recognized as an inflammatory condition, in which menstrual disturbances trigger the production of leukocytes and inflammatory mediators [50]. Leukocyte infiltration promotes the release of pro-inflammatory cytokines, including *TNF-α* and *IL-6*, leading to endometrial edema and hemorrhage [51]. This study showed that DBD effectively reduced the writhing times and prolonged the writhing latency in primary dysmenorrhea female mice. Concurrently, DBD downregulated mRNA expression of inflammation-related genes, including *Tnfa* and *Il6*. Histopathological analysis further confirmed that DBD alleviated primary dysmenorrhea-induced inflammation and tissue edema.

Primary dysmenorrhea leads to increased production of PGF2α, which also plays a significant role in inflammatory responses [52]. Moreover, PGF2α may sensitize peripheral nerve endings, lowering the pain threshold [53]. Clinical studies have shown elevated PGF2α levels in patients with primary dysmenorrhea compared to healthy individuals [54,55]. The present study confirms that an OT-induced primary dysmenorrhea model increases PGF2α levels. In contrast, DBD significantly suppressed primary dysmenorrhea-induced PGF2α elevation. PGF2α is the endogenous ligand of PTGFR, and their binding triggers uterine smooth muscle contraction [56]. PTGFR, a member of the GPCR family, is highly expressed in smooth muscle and the uterine myometrium [57]. Upon GPCR activation, calcium ions are released from the sarcoplasmic reticulum via the IP_3_/Ca^2+^ pathway [58,59]. Experimental results demonstrate that DBD not only reduces levels of PGF2α and PTGFR but also decreases concentrations of Ca^2+^ and IP_3_.

Quercetin, a representative flavonoid, exhibits significant pharmacological effects in the treatment of various gynecological conditions, such as polycystic ovary syndrome, premature ovarian failure, endometriosis, endometrial cancer, and ovarian cancer [60]. Preliminary experiments on isolated uterine tissue indicate that Quercetin exhibits a more potent uterine-relaxant effect compared to other compounds. Among the eight active components, Quercetin exhibited the most favorable binding affinity with PTGFR in molecular docking analysis, with the lowest binding energy. These findings suggest that Quercetin may be the key constituent in DBD responsible for uterine smooth muscle relaxation and the treatment of primary dysmenorrhea. The therapeutic potential of Quercetin was further evaluated in a mouse model of primary dysmenorrhea. Treatment with Quercetin not only alleviated pain symptoms but also reduced Ca^2+^ and IP_3_ concentrations. These findings support Quercetin as a key active constituent in DBD for the treatment of primary dysmenorrhea.

Collectively, our findings offer theoretical support for understanding how DBD and its active constituents relax the uterus and treat primary dysmenorrhea. However, several limitations remain. Firstly, experiments were conducted solely at the in vitro tissue and animal levels. To further validate the IP_3_/Ca^2+^ pathway mechanism, we will establish PTGFR-overexpressing cell lines to detect alterations in downstream calcium signaling. Although this study demonstrated that eight compounds from DBD possess uterine-relaxant effects, it remains unclear whether these effects are attributable to the dominant action of a specific molecular family or to synergistic interactions among multiple molecules. In subsequent studies, we will employ network pharmacology to predict potential target molecules and integrate multi-omics approaches, such as transcriptomics and metabolomics, to elucidate the synergistic regulatory mechanisms involving multiple targets and pathways at a systemic level. This will offer a more comprehensive theoretical foundation for the application of DBD in the treatment of primary dysmenorrhea.

## 4. Materials and Methods

### 4.1. Chemicals and Reagents

Quercetin, Formononetin, Ononin, Ferulic acid, Senkyunolide I, Calycosin, Ligustilide and Calycosin-7-O-β-D-glucoside were purchased from Chengdu Pusi Biotechnology Co., Ltd. (Chengdu, China). AR and AES were provided by the Affiliated Hospital of Chengdu University of Traditional Chinese Medicine (Chengdu, China), and their quality was verified according to the Chinese Pharmacopoeia (2025 edition). Nifedipine was purchased from Shanghai McLean Biochemical Technology Co., Ltd. (Shanghai, China). Oxytocin was purchased from Shenggong Biotechnology (Shanghai) Co., Ltd. (Shanghai, China). 2-APB was purchased from Medchemexpress LLC. (Shanghai, China). Estradiol Benzoate Injection was purchased from Shanghai Quanyu Biotech Animal Pharmaceutical Co., Ltd. (Shanghai, China). The remaining reagents were analytical grade from domestic suppliers.

### 4.2. Preparation of DBD

AES (60 g) and AR (300 g) were soaked in 8 volumes of water (*w*/*v*) for 30 min and then decocted for 90 min. The decoction was filtered through muslin. The residue was mixed with 6 volumes of water and decocted for another 90 min. The combined filtrates were concentrated on a rotary evaporator at 60 °C. The concentrate was then lyophilized to obtain 136 g of DBD lyophilized powder, yielding 37.8% (1 g of DBD lyophilized powder corresponds to 2.65 g of crude DBD herbal material). The powder was stored at −20 °C until use. AES and AR were prepared separately following the same procedure.

### 4.3. Animals

Female C57BL/6 mice, aged 6–8 weeks, were purchased from GemPharmatech Co., Ltd. (Nanjing, China) (SCXK (chuan) 2020-0034). Drinking water and standard chow were provided without restriction. The female mice were accommodated in plastic cages under standardized laboratory conditions: humidity 60–80%, temperature 22 ± 2 °C, and a 12 h light/dark cycle. This study was reviewed and approved by the Animal Ethics Committee of Chengdu University of Traditional Chinese Medicine (Approval No. 2024035).

### 4.4. Qualitive Analysis of Constituents in DBD Extract by UPLC–Orbitrap MS Technology

The DBD was diluted to 0.5 g/mL with methanol, then centrifuged at 12,000 rpm for 10 min. It was then filtered through a 0.22 μm syringe filter. A UHPLC system (Hypersil GOLD column, 100 × 2.1 mm, 1.9 µm; Thermo Scientific, Waltham, MA, USA) was used for compound separation. The mobile phase consisted of water with 0.1% formic acid (A) and acetonitrile (B), flowing at 0.3 mL/min. The column temperature was maintained at 40 °C, the injection volume was 1 µL, and the autosampler temperature was set to 8 °C. The following gradient was applied: 0–0.5 min, 2% B; 0.5–12 min, 2% to 50% B; 12–14 min, 50% to 98% B; 14–16 min, 98% B; 16–16.1 min, 98% to 2% B; 16.1–18 min, 2% B. Data were acquired on an Orbitrap Exploris 120 high-resolution mass spectrometer (Thermo Scientific, USA) using a heated electrospray ionization (H-ESI) source. Prior to analysis, mass calibration was performed using standard solutions delivered by a syringe pump (SKE10, Chemyx, Houston, TX, USA) equipped with a microsyringe (1750RNR 500 µL SYR, Hamilton, Reno, NV, USA), ensuring that mass errors for characteristic ions were below 5 ppm. MS parameters were optimized as follows: spray voltage, +3.2 kV (positive) and −3.0 kV (negative); vaporizer, 350 °C; ion transfer tube, 320 °C; sheath gas flow rate, 40 Arb; auxiliary gas flow rate, 10 Arb. Full MS scans were acquired at a resolution of 60,000 over an *m*/*z* range of 70–1050. The RF lens voltage was set to 70%, and the automatic gain control (AGC) target was set to standard mode. Data-dependent MS/MS acquisition was performed at a resolution of 15,000 with stepped HCD collision energies: 20, 40, and 60%. Data processing was performed using Xcalibur software (version 4.6 Thermo Scientific, USA) and compounds were identified by matching the accurate mass-to-charge ratios against local and online chemical databases.

### 4.5. Preparation of Uterine Muscle Strips

Female C57BL/6 mice were administered estradiol benzoate (10 mg/kg) via intraperitoneal injection for three consecutive days [61,62]. The animals were euthanized by decapitation. Uterine tissues were promptly excised and placed in pre-chilled Krebs-Henseleit (K-H) solution (composition in mM: NaCl 118, KCl 4.7, CaCl_2_ 2.5, KH_2_PO_4_ 1.2, MgCl_2_·6H_2_O 1.2, NaHCO_3_ 25, glucose·H_2_O 11, HEPES 5), which was continuously aerated with carbogen (95% O_2_/5% CO_2_). After carefully removing the surrounding adipose and connective tissues, uterine muscle strips (approximately 0.5 cm in length) were isolated and suspended in bath containing K-H solution maintained at approximately 37 °C and continuously aerated with carbogen (95% O_2_/5% CO_2_). The uterine muscle strips were initially stretched to a tension of 0.5 g and then allowed to equilibrate for at least 30 min until stable spontaneous contractions developed [63] (Figure S1A). Following a 30 min equilibration period, uterine muscle strips were first contracted with KCl (60 mM). The uterine muscle strips were then washed three times with K-H solution at 10 min intervals, and a second contraction was induced with KCl (60 mM). Uterine muscle strips exhibiting less than 10% difference in amplitude between the two KCl (60 mM)-induced contractions were used for subsequent experiments. At the end of each experiment, the uterine muscle strips were rinsed three times with K-H solution at 10 min intervals. A final challenge with KCl (60 mM) or OT (50 ng/mL) was then applied. Recurrence of contraction confirmed that the strips remained viable, indicating that any observed reduction in contractility was attributable to drug treatment rather than cytotoxicity [64] (Figure S1B,C). Changes in tension were recorded using a PowerLab multifunctional physiological acquisition system.

### 4.6. Assessment of Drug-Induced Uterine Contractions

The uterine muscle strips were then exposed to either OT (50 ng/mL) or KCl (60 mM) [65] for approximately 15 min to induce a contractile response. Subsequently, increasing concentrations of DBD, its constituent herbs (AES and AR), or active constituents (Quercetin, Formononetin, Ononin, Ferulic acid, Senkyunolide I, Calycosin, Ligustilide, Calycosin-7-O-β-D-glucoside) were cumulatively added to the organ bath. Each concentration was allowed to act for approximately 10 min before the next addition. Changes in uterine muscle strips tension were continuously recorded throughout the experiment.

### 4.7. Effect of DBD and Its Active Constituents on Ca^2+^-Dependent Contractions

Following equilibration for over 30 min to achieve stable contractions, uterine muscle strips were exposed to Ca^2+^-free K-H solution for 10 min. Subsequently, increasing concentrations of DBD, or active constituents (Quercetin, Calycosin, Ligustilide) were cumulatively added to the organ bath. Following a 10 min incubation, contractility was restored by the cumulative addition of CaCl_2_ solutions at increasing concentrations (0.5–10 mM) [66,67,68].

### 4.8. The Effect of DBD and Its Active Constituents on Extracellular Calcium Influx

KCl (60 mM) was used to elicit pre-contraction in uterine muscle strips. After the contraction reached a plateau phase, 5 nM Nifedipine (the KCl-induced response was attenuated without significantly affecting contraction amplitude) was introduced [65]. Following approximately 20 min of incubation with the blocker, increasing concentrations of DBD, Quercetin, Calycosin, or Ligustilide were cumulatively added. Changes in uterine muscle strip tension were subsequently recorded.

### 4.9. The Effect of DBD and Its Active Constituents on Intracellular Calcium Release

After the uterine muscle strip has equilibrated, the bath solution was replaced with KCl (60 mM) solution. This solution was maintained for 15 min to promote calcium ion transport into the sarcoplasmic reticulum. Following this, the tissue was exposed to a Ca^2+^-free solution containing EDTA (0.3 mM) for 15 min. Subsequent addition of OT (50 ng/mL) induced contractions of the uterine muscle strips. After the contractile response stabilized, increasing concentrations of DBD, Quercetin, Calycosin, or Ligustilide were cumulatively added. Changes in uterine muscle strip tension were subsequently recorded. Additionally, the mechanism of intracellular calcium release was preliminarily investigated using the IP_3_R antagonist 10 μM 2-APB [69].

### 4.10. Oxytocin-Induced Writhing Test

We conducted the oxytocin-induced writhing test based on previously reported methods [70,71,72]. Seven experimental groups of female mice (n = 6 each) were established: a control group, a model group, three groups receiving DBD at low (DBD-L, 3.78 g/kg), medium (DBD-M, 7.56 g/kg), or high (DBD-H, 15.12 g/kg) doses, a positive control group (TJB, 3 g/kg) [73], and a Quercetin group (50 mg/kg) [74]. From the first day of the experiment, mice in the DBD groups, TJB group, and Quercetin group received oral administration once daily for 11 consecutive days; mice in the control and model groups were given an equivalent volume of deionized water. Starting from day 8, all groups except the control received estradiol benzoate (1 mg/kg i.p. for 3 days). One hour after the final oral administration on day 11, all groups except the control were injected intraperitoneally with 2 IU OT per female mouse. Writhes were counted for 30 min post-OT injection. A complete writhing response was characterized by abdominal retraction and concavity, extension of the hind limbs, pressing of the lower abdomen against the cage floor, and elevation of the hindquarters [71].

### 4.11. Biochemical Analysis

Intracellular Ca^2+^ levels in uterine tissues were assayed using a commercial kit (Nanjing Jiancheng Bioengineering Institute, Nanjing, China). Quantitative analysis of PLC and IP_3_ was performed using ELISA kits (Wuhan Elabscience Biotechnology Co., Ltd., Wuhan, China), and all assays were performed in compliance with the manufacturer’s recommendations.

### 4.12. Histological Analysis

Following fixation in 4% paraformaldehyde (>24 h), uterine tissues were dehydrated and embedded in paraffin. Serial sections, cut at a thickness of 3–5 μm, were prepared and stained with hematoxylin and eosin (HE) according to routine protocols. The stained sections were subsequently imaged using a Pannoramic SCAN II scanner for histomorphological analysis.

### 4.13. Real-Time qPCR

Total RNA was isolated from uterine tissues by homogenization in TRIzol reagent. Following the manufacturer’s guidelines, the PrimeScript™ FAST RT reagent kit (Takara, Kusatsu, Japan) was employed to reverse transcribe the purified RNA into cDNA. A real-time PCR analysis was conducted on a real-time PCR detection system employing TB Green^®^ Premix Ex Taq™ II (Takara, Kusatsu, Japan), in accordance with the manufacturer’s recommended procedures. Gene expression levels were quantified by the 2^−ΔΔCt^ method, with *Actb* mRNA serving as the endogenous reference. Primer sequences are shown in Table 3.

### 4.14. Statistical Data

All values are given as mean ± standard error of the mean (SEM). Statistical evaluation was performed with GraphPad Prism (version 8.0). Relaxant responses were expressed as a percentage of the maximal contractile tension induced by KCl (60 mM) or OT (50 ng/mL), which was set as 100%. The half-maximal effective concentration (EC_50_) was determined by non-linear regression analysis of cumulative concentration–response curves. EC_50_ units were used (mg/mL for extracts, μM for compounds). Differences between the two groups were analyzed using Student’s *t*-test, while comparisons among multiple groups were performed by one-way ANOVA followed by Dunnett’s post hoc test for pairwise comparisons. Statistical significance was set at *p* < 0.05.

## 5. Conclusions

DBD and its active constituents relax the uterus by inhibiting L-type calcium channels and the IP_3_/Ca^2+^ pathway, thereby treating primary dysmenorrhea.

## Figures and Tables

**Table 1:** Identification of compounds in DBD by UPLC–Orbitrap MS.

No.	RT (min)	Compounds	Formula	Ion Mode	*m*/*z*	Error	MS/MS Fragment Ions	Source
1	0.73	Canavanine	C_5_H_12_N_4_O_3_	[M + H]^+^	177.0980	−0.49	160.0716, 118.0498, 102.0548, 76.0504	AR
2	0.76	Arginine	C_6_H_14_N_4_O_2_	[M + H]^+^	175.1190	−0.07	158.0926, 130.0976, 116.0706, 70.0652, 60.0557	AES, AR
3	0.80	Aspartic acid	C_4_H_7_NO_4_	[M − H]^−^	132.0300	+1.04	115.0037, 88.0403	AES, AR
4	0.86	Inosine	C_10_H_12_N_4_O_5_	[M − H]^−^	267.0720	−0.53	249.0620, 113.0245, 99.0087	AES
5	0.89	Raffinose	C_18_H_32_O_16_	[M + H]^+^	505.1770	+0.84	-	AES, AR
				[M − H]^−^	503.1620	+1.02	323.0988, 221.0666, 179.0561, 89.0245	AES, AR
6	0.90	Malic acid	C_4_H_6_O_5_	[M − H]^−^	133.0141	+0.96	115.0037, 89.0244, 71.0139	AES, AR
7	0.90	Adenine	C_5_H_5_N_5_	[M + H]^+^	136.0618	+0.06	119.0353	AES, AR
8	0.94	Sucrose	C_12_H_22_O_11_	[M + H]^+^	343.1236	+0.33	163.0604, 145.0497, 127.0391, 97.0286, 85.0285	AES, AR
				[M − H]^−^	341.1085	+0.65	179.0561, 119.0350, 101.0244, 89.0244	AES, AR
9	0.96	Mycose	C_12_H_22_O_11_	[M + H]^+^	343.1236	+0.24	163.0604, 145.0497, 127.0391, 97.0286, 85.0285, 69.0336	AES, AR
10	0.98	Citric acid	C_6_H_8_O_7_	[M + H]^+^	193.0347	+2.18	-	AES, AR
				[M − H]^−^	191.0195	+0.90	129.0194, 111.0088, 87.0088	AES, AR
11	1.31	Tyrosine	C_9_H_11_NO_3_	[M + H]^+^	182.0811	−0.22	165.0547, 147.0442, 136.0758, 123.0441, 91.0452	AES, AR
				[M − H]^−^	180.0665	+0.98	163.0401, 119.0503, 93.0346	AES, AR
12	1.32	Adenosine	C_10_H_13_N_5_O_4_	[M + H]^+^	268.104	0.00	136.0618	AES, AR
13	1.35	Guanosine	C_10_H_13_N_5_O_5_	[M + H]^+^	284.0989	0.00	152.0567	AES, AR
14	1.36	Guanine	C_5_H_5_N_5_O	[M + H]^+^	152.0567	+0.02	135.0301, 110.0347	AES, AR
15	1.56	Leucine	C_6_H_13_NO_2_	[M + H]^+^	132.1019	−0.27	86.0964	AES, AR
				[M − H]^−^	130.0872	+0.95	88.0405	AES, AR
16	1.68	Xanthosine	C_10_H_12_N_4_O_6_	[M + H]^+^	285.0831	+0.38	-	AR
17	1.69	Arg-Ile	C_12_H_25_N_5_O_3_	[M + H]^+^	288.2030	+0.05	271.1767, 175.1190, 70.0651	AES, AR
18	2.10	Piscidic acid	C_11_H_12_O_7_	[M + H]^+^	257.0658	+0.86	-	AR
				[M − H]^−^	255.0509	+0.99	193.0502, 179.0349, 165.0557, 149.0604	AR
19	2.65	Phenylalanine	C_9_H_11_NO_2_	[M + H]^+^	166.0862	−0.09	120.0807	AES, AR
20	3.07	Pantothenate	C_9_H_17_NO_5_	[M + H]^+^	220.1180	+0.00	202.1077, 184.0968, 124.0757, 90.0549	AES, AR
21	3.09	Succinoadenosine	C_14_H_17_N_5_O_8_	[M + H]^+^	384.1150	+0.00	252.0727, 192.0516, 162.0774, 136.0617	AES, AR
22	3.43	Pyrocatechuic acid	C_7_H_6_O_4_	[M − H]^−^	153.0193	+1.06	-	AR
23	3.95	Vanillin	C_8_H_8_O_3_	[M − H]^−^	151.0400	+1.01	123.0451, 121.0295, 107.0502	AES
24	3.97	Tryptophan	C_11_H_12_N_2_O_2_	[M + H]^+^	205.0971	−0.31	188.0706, 146.0600, 118.0650	AES
25	3.97	3-Indoleacrylic acid	C_11_H_9_NO_2_	[M + H]^+^	188.0705	−0.39	170.0597, 146.0600, 118.0650	AES, AR
26	3.98	Indole-3-carboxaldehyde	C_9_H_7_NO	[M + H]^+^	146.0600	+0.00	118.0651, 100.0757, 91.0545	AES, AR
27	4.22	Pratensein-Glc-Glc	C_28_H_32_O_16_	[M + H]^+^	625.1766	+0.41	463.1219, 301.0706	AR
28	4.24	Complanatuside	C_28_H_32_O_16_	[M + H]^+^	625.1765	+0.22	301.0706	AR
29	4.46	4-Hydroxybenzoic acid	C_7_H_6_O_3_	[M + H]^+^	139.0389	−0.22	121.0284	AR
30	4.58	Chlorogenic acid isomer	C_16_H_18_O_9_	[M + H]^+^	355.1025	+0.51	163.0390, 135.0441	AES, AR
				[M − H]^−^	353.0876	+0.88	191.0560, 179.0349, 135.0452	AES, AR
31	4.60	Chlorogenic acid	C_16_H_18_O_9_	[M − H]^−^	353.0876	+0.84	191.056	AES, AR
32	4.61	Umbelliferone	C_9_H_6_O_3_	[M + H]^+^	163.0390	+0.12	145.0284, 135.0440, 117.0334	AES
33	4.64	Guaiacol	C_7_H_8_O_2_	[M + H]^+^	125.0597	−0.21	107.0491, 97.0284	AES
				[M − H]^−^	123.0451	+1.03	105.7571, 95.0139	AES
34	4.65	Chlorogenic acid	C_16_H_18_O_9_	[M + H]^+^	355.1024	+0.26	163.039	AES, AR
35	4.65	Sinapic acid	C_11_H_12_O_5_	[M + H]^+^	225.0757	−0.09	-	AES
36	4.66	Chlorogenic acid isomer	C_16_H_18_O_9_	[M + H]^+^	355.1024	+0.26	163.039	AES, AR
37	4.73	Chlorogenic acid isomer	C_16_H_18_O_9_	[M − H]^−^	353.0876	+0.91	191.0560, 179.0349, 173.0455, 135.0452	AES, AR
38	5.01	Phthalic anhydride	C_8_H_4_O_3_	[M + H]^+^	149.0233	+0.00	131.0855, 121.0284, 91.0541, 65.0386	AES
39	5.04	Caffeic acid	C_9_H_8_O_4_	[M + H]^+^	181.0495	+0.03	135.0918	AES, AR
				[M − H]^−^	179.0349	+0.99	135.0452	AES, AR
40	5.19	Vanillic acid	C_8_H_8_O_4_	[M + H]^+^	169.0495	+0.09	151.0390, 123.0807, 79.0541	AES
				[M − H]^−^	167.0348	+0.91	123.0452	AES
41	5.21	Icariside F2	C_18_H_26_O_10_	[M − H]^−^	401.1452	+1.00	269.1030, 161.0455, 113.0244, 101.0244	AES
42	5.29	Riboflavin	C_17_H_20_N_4_O_6_	[M + H]^+^	377.1456	+0.18	234.0875, 216.0767, 172.0868	AR
43	5.55	Rhamnocitrin 3-Oglucoside	C_22_H_22_O_11_	[M − H]^−^	461.1091	+1.29	299.0560, 284.0324, 255.0299	AR
44	5.57	Aspartic acid	C_4_H_7_NO_4_	[M + H]^+^	134.0448	−0.11	116.0645	AES, AR
45	5.64	Pratensein-7-O-β-D-glucoside	C_22_H_22_O_11_	[M + H]^+^	463.1236	+0.31	301.0706, 286.0480, 241.0500, 213.0547	AR
				[M − H]^−^	461.1091	+1.23	299.0560, 284.0324, 255.0299	AR
46	5.75	5-Feruoylquinic acid	C_17_H_20_O_9_	[M − H]^−^	367.1037	+1.31	191.0560, 173.0454, 134.0373, 93.0346	AES
47	5.96	(+) Lariciresinol-4′-O-β-D-Apiofuranosyl-(1-2)-β-D-Glucopyranosyl	C_31_H_42_O_15_	[M − H]^−^	653.2451	+1.12	623.2345, 329.1394	AR
48	6.07	Vanillin	C_8_H_8_O_3_	[M + H]^+^	153.0546	+0.06	125.0597, 111.0440, 93.0334, 65.0386	AES
49	6.10	Sinapic acid	C_11_H_12_O_5_	[M − H]^−^	223.0611	+0.97	208.0376, 193.0142, 149.0246	AR
50	6.19	Rhamnocitrin 3-neohesperidoside isomer	C_28_H_32_O_15_	[M + H]^+^	609.1813	−0.09	285.0758, 270.0523	AR
				[M − H]^−^	607.1669	+1.18	413.1089, 283.0605, 193.0505, 137.0244	AR
51	6.25	Dimethyl azelate	C_11_H_20_O_4_	[M + H]^+^	217.1434	−0.03	-	AES
52	6.35	Sissotrin	C_22_H_22_O_10_	[M + H]^+^	447.1284	−0.41	-	AR
53	6.49	Liquiritin	C_21_H_22_O_9_	[M − H]^−^	417.1192	+1.19	255.0663, 135.0088, 119.0503	AR
54	6.53	Calycosin-7-O-β-D-glucoside	C_22_H_22_O_10_	[M + H]^+^	447.1285	−0.21	285.0757, 270.05231, 253.04971, 225.05463, 137.02345	AR
55	6.53	Ferulic acid	C_10_H_10_O_4_	[M − H]^−^	193.0505	+0.96	178.0272, 149.0609, 134.0374	AES, AR
56	6.54	Isoferulic Acid	C_10_H_10_O_4_	[M − H]^−^	193.0505	+0.98	178.0272, 149.0609, 134.0374	AES, AR
57	6.56	Senkyunolide I	C_12_H_16_O_4_	[M + H]^+^	225.1121	−0.20	207.1015, 165.0910	AES
58	6.57	N-Acetylphenylalanine	C_11_H_13_NO_3_	[M + H]^+^	208.0968	+0.00	-	AES, AR
				[M − H]^−^	206.0822	+1.00	164.0717, 147.0452, 91.0554	AES, AR
59	6.57	7-Methoxycoumarin isomer	C_10_H_8_O_3_	[M + H]^+^	177.0546	−0.06	149.0597, 145.0284, 117.0334, 89.0385	AES
60	6.57	Dimethyl phthalate	C_10_H_10_O_4_	[M + H]^+^	195.0652	+0.02	177.0546, 171.1538, 145.0284, 117.0334	AES
				[M − H]^−^	193.0505	+0.96	178.0272, 149.0609, 134.0374	AES
61	6.58	7-Methoxycoumarin isomer	C_10_H_8_O_3_	[M + H]^+^	177.0546	+0.05	149.0597, 145.0284, 117.0334, 89.0385	AES
62	6.69	Tamarixin	C_22_H_22_O_12_	[M − H]^−^	477.1039	+1.19	315.0457, 271.0230	AR
63	6.70	N-Acetyltryptophan	C_13_H_14_N_2_O_3_	[M − H]^−^	245.0931	+0.99	203.0827, 116.0353, 98.0248, 74.0248	AES, AR
64	6.72	Apigenin-7-O-β-D-glucoside	C_21_H_20_O_10_	[M + H]^+^	433.1131	+0.48	332.1817, 271.0602	AR
				[M − H]^−^	431.0982	+0.91	269.0457, 239.0342, 211.1095	AR
65	6.96	Apigenin-5-O-β-D-glucopyranoside	C_21_H_20_O_10_	[M − H]^−^	431.0986	+3.08	385.1786, 268.0376, 239.0352, 205.0870	AR
66	7. 00	1,3- Dicaffeoylquinic acid	C_25_H_24_O_12_	[M − H]^−^	515.1195	+1.11	353.0879, 335.0711, 191.0559, 179.0349, 173.0454, 135.0451	AES
67	7.03	3′-methoxy-5′-hydroxy-isoflavaone-7-O-β-Dglucopyranoside	C_22_H_22_O_10_	[M − H]^−^	445.1140	+1.12	283.0609, 281.0454, 268.0374, 253.0505, 239.0348	AR
68	7.11	(+)-7-epi-Syringaresinol 4′-glucoside	C_28_H_36_O_13_	[M − H]^−^	579.2081	+0.85	417.1555, 387.1084, 353.1025, 181.0506, 166.0270, 151.0036	AR
69	7.19	Genistin	C_21_H_20_O_10_	[M − H]^−^	431.0986	+1.33	268.0376, 239.0352, 205.0870	AR
70	7.19	9-(2,3-dihydroxypropoxy)-9-oxononanoic acid	C_12_H_22_O_6_	[M − H]^−^	261.1342	+0.96	187.0976, 169.0867, 125.0972	AR
71	7.19	Isomucronulatol-7,2′-di-glucoside	C_29_H_38_O_15_	[M − H]^−^	625.2138	+1.11	463.1611, 301.1082	AR
72	7.19	Isomucronulatol-7,2′-di-glucoside isomer	C_29_H_38_O_15_	[M − H]^−^	625.2138	+1.11	463.1611, 301.1082	AR
73	7.29	Calycosin-7-O-β-Dglucoside-6″-Omalonate	C_25_H_24_O_13_	[M + H]^+^	533.1290	+0.01	285.0755, 270.0522	AR
74	7.34	4′-methoxykaempferol-3-O-β-Dglucoside	C_22_H_22_O_11_	[M + H]^+^	463.1235	+0.03	301.0706, 286.0473, 241.0498, 213.0542	AR
75	7.39	Licoagroside D or isomer	C_22_H_24_O_10_	[M − H]^−^	447.1299	+1.28	285.0768, 270.0533	AR
76	7.41	Licoagroside D or isomer	C_22_H_24_O_10_	[M − H]^−^	447.1298	+1.22	285.0768, 270.0533	AR
77	7.48	Hesperetin 7-Oglucoside	C_22_H_24_O_11_	[M − H]^−^	463.1248	+1.30	301.0721, 191.0349	AR
78	7.56	1,4-Dicaffeoylquinic acid	C_25_H_24_O_12_	[M − H]^−^	515.1196	+1.17	353.0879, 191.0559, 179.0349, 173.0454	AES
79	7.65	Azelaic acid	C_9_H_16_O_4_	[M − H]^−^	187.0974	+0.92	169.0871, 143.1078, 125.0973	AES, AR
80	7.66	7-Methoxycoumarin	C_10_H_8_O_3_	[M − H]^−^	175.0402	+1.22	160.0167, 132.0218	AES
81	7.69	Senkyunolide S	C_12_H_16_O_5_	[M − H]^−^	239.0924	+1.02	195.1026, 154.0272, 111.0452, 101.0608	AES
82	7.76	Z-6,7-epoxyligustilide	C_12_H_14_O_3_	[M + H]^+^	207.1015	−0.10	189.0912, 161.0961, 119.0854	AES
83	7.82	Salicylic acid	C_7_H_6_O_3_	[M − H]^−^	137.0242	+0.93	93.0346	AR
84	7.82	4-Hydroxybenzoic acid	C_7_H_6_O_3_	[M − H]^−^	137.0242	+0.94	93.0346	AR
85	7.97	Calycosin-7-O-β-D-(6″-O-acetyl)-glucoside	C_24_H_24_O_11_	[M + H]^+^	489.1392	+0.11	285.0756, 270.0523, 225.0547	AR
86	7.98	Ononin-Glc	C_28_H_32_O_14_	[M + H]^+^	593.1865	+0.06	269.0809, 254.0567, 213.0916	AR
87	8.00	Glycyroside	C_27_H_30_O_13_	[M + H]^+^	563.1761	+0.31	269.0807, 254.0574	AR
				[M − H]^−^	561.1611	+0.86	267.0661, 252.0427	AR
88	8.13	Unknown	C_28_H_34_O_14_	[M − H]^−^	593.1877	+1.19	505.1718, 417.1558, 402.1324, 387.1083,181.0506, 166.0271	AR
89	8.14	Senkyunolide F	C_12_H_14_O_3_	[M + H]^+^	207.1015	−0.49	189.0912, 161.0961, 119.0854	AES
90	8.17	Pratensein	C_16_H_12_O_6_	[M − H]^−^	299.0561	+1.11	284.0329, 255.0301	AR
91	8.25	Ononin	C_22_H_22_O_9_	[M + H]^+^	431.1333	−0.76	269.0807, 254.0573	AR
				[M − H]^−^	429.1190	+1.04	-	AR
92	8.32	Afrormosin 7-O-glucoside	C_23_H_24_O_10_	[M + H]^+^	461.1444	+0.34	299.0912	AR
93	8.33	Daidzein	C_15_H_10_O_4_	[M + H]^+^	255.0652	+0.06	199.0753, 137.0232	AR
				[M − H]^−^	253.0505	+0.99	225.0552	AR
94	8.39	3-Hydro-9,10-diMPPen-Glc	C_28_H_34_O_14_	[M − H]^−^	593.1877	+1.19	299.0923, 284.0689, 269.0453	AR
95	8.49	6,7-Epoxyligustilide	C_12_H_14_O_3_	[M + H]^+^	207.1015	−0.54	189.0912, 161.0961	AES
96	8.56	Liquiritigenin	C_15_H_12_O_4_	[M + H]^+^	257.0808	+0.02	147.0441, 137.0233, 119.0493	AR
97	8.71	Methylinissolin-3-O-β-D-glucoside	C_23_H_26_O_10_	[M + H]^+^	463.1599	+0.01	301.1073, 269.0808, 167.0703	AR
98	8.71	Methylnissolin	C_17_H_16_O_5_	[M + H]^+^	301.1070	−0.29	269.0811, 167.0703	AR
				[M − H]^−^	299.0924	+0.98	284.0691, 269.0455, 241.0508, 158.0572	AR
99	8.76	Prunin	C_21_H_22_O_10_	[M − H]^−^	433.1140	+1.12	271.0611, 256.0375, 243.0663	AR
100	8.80	Calycosin	C_16_H_12_O_5_	[M + H]^+^	285.0756	−0.56	270.0525, 253.0498, 225.0544	AR
				[M − H]-	283.0609	+0.81	268.0377, 211.0401	AR
101	8.80	5,7-Dihydroxy-4-methoxyisoflavone	C_16_H_12_O_5_	[M + H]^+^	285.0756	−0.56	270.0525, 253.0498, 225.0544, 137.0231	AR
102	8.95	Isomucronulatol-7-O-β-D-glucoside	C_23_H_28_O_10_	[M + H]^+^	465.1756	+0.10	303.1219, 167.0706, 123.0440	AR
				[M − H]^−^	463.1610	+1.17	301.1084, 286.0849, 271.0615, 179.0717, 164.0479, 135.0452	AR
103	9.01	3,9-dihydroxyligustilide	C_12_H_16_O_4_	[M − H]^−^	223.0975	+1.04	179.1077, 137.0972, 95.0503	AES
104	9.03	Formononetin-7-O-β-D-glucoside-6″-Omalonate	C_25_H_24_O_12_	[M + H]^+^	517.1339	−0.28	269.0807, 254.0573	AR
105	9.03	Formononetin-7-O-β-D-glucoside-6″-Omalonate isomer	C_25_H_24_O_12_	[M + H]^+^	517.1339	−0.28	269.0807, 254.0573	AR
106	9.03	3,4-Dicaffeoylquinic acid	C_25_H_24_O_12_	[M − H]^−^	515.1196	+1.23	353.0879, 335.0711, 191.0559, 179.0349, 173.0454, 135.0451	AES
107	9.06	Rhamnocitrin 3-Oglucoside	C_22_H_22_O_11_	[M + H]^+^	463.1236	+0.18	301.0706, 231.0652, 167.0342	AR
108	9.08	4′-methoxykaempferol-3-O-β-Dglucoside	C_22_H_22_O_11_	[M − H]^−^	461.1090	+1.20	299.0562, 165.0193	AR
109	9.29	Senkyunolide H-7-Acetate	C_14_H_18_O_5_	[M − H]^−^	265.1081	+1.07	247.1340, 221.1182	AES
110	9.30	Dihydrocapsaicin	C_18_H_29_NO_3_	[M + H]^+^	308.2220	−0.00	290.2120, 262.2169, 179.1305	AR
111	9.41	Senkyunolide J or isomer	C_12_H_18_O_4_	[M − H]^−^	225.1131	+0.96	207.1027, 181.1234, 163.1130	AES
112	9.55	Astragaloside IV, V, VI or VII	C_47_H_78_O_19_	[M + H]^+^	947.5213	+0.31	569.3848, 455.3523, 437.3412, 419.3307, 143.1067	AR
113	9.59	Isomucronulatolacetyl-Glc	C_25_H_30_O_11_	[M − H]^−^	505.1714	+1.01	445.1506, 301.1081, 271.0613, 121.0295	AR
114	9.65	glucoside-6″-Omalonate	C_26_H_28_O_13_	[M + H]^+^	549.1604	+0.24	301.1077, 167.0704	AR
115	9.73	Genistein	C_15_H_10_O_5_	[M + H]^+^	271.0601	+0.07	229.0858, 153.0182, 121.0284	AR
116	9.83	Senkyunolide D	C_12_H_14_O_4_	[M − H]^−^	221.0818	+0.96	177.092	AR
117	9.87	Afrormosin	C_17_H_14_O_5_	[M + H]^+^	299.0914	−0.10	283.0600, 266.0573, 255.0655, 237.0546	AR
118	9.87	6″-O-acetylononin	C_24_H_24_O_10_	[M + H]^+^	473.1442	−0.05	269.0808	AR
119	9.97	Pendulone	C_17_H_16_O_6_	[M + H]^+^	317.1019	−0.11	299.0912, 289.1075, 183.0650, 163.0390, 135.0440, 107.0490	AR
				[M − H]^−^	315.0875	+1.18	285.0396, 109.0295	AR
120	10.04	Kumatakenin	C_17_H_14_O_6_	[M − H]^−^	313.0719	+1.21	298.0484, 283.0248, 255.0300, 227.0350	AR
121	10.16	Pratensein	C_16_H_12_O_6_	[M + H]^+^	301.0707	+0.05	269.0448, 241.0496, 167.0703	AR
122	10.17	9,12,13-Trihydroxyoctadeca-10,15-dienoic acid	C_18_H_32_O_5_	[M − H]^−^	327.2176	+1.02	309.2069, 291.1967, 229.1447, 211.1340, 171.1027	AR
123	10.17	7-Methyl-kaempferol	C_16_H_12_O_6_	[M + H]^+^	301.0707	+0.05	286.0472, 269.0448, 241.0496, 167.0703	AR
				[M − H]^−^	299.0561	+1.11	284.0329, 255.0301	AR
124	10.19	Methylnissolin-3-O-β-D-(6′-O-acetyl)-glucoside	C_25_H_28_O_11_	[M + H]^+^	505.1704	−0.06	301.1070, 167.0703	AR
125	10.20	3,7,8-trihydroxy-4-methoxyisoflavone	C_16_H_12_O_6_	[M − H]^−^	299.0561	+1.11	2840329, 267.0291, 256.0370, 255.0307	AR
126	10.50	9,10,11-Trihydroxyoctadeca-12,15-dienoic acid	C_18_H_32_O_5_	[M − H]^−^	327.2177	+1.14	309.2069, 291.1967, 229.1447, 211.1340, 183.1390, 171.1027	AR
127	10.79	9,10,13-trihydroxy-11-octadecenoic acid	C_18_H_34_O_5_	[M − H]^−^	329.2332	+0.97	229.1445, 211.1340, 171.1027	AR
128	10.93	(Z)-5,8,11-trihydroxyoctadec-9-enoic acid	C_18_H_32_O_5_	[M − H]^−^	329.2332	+0.93	291.1967, 229.1447, 211.1340, 171.1027	AR
129	10.95	Formononetin	C_16_H_12_O_4_	[M + H]^+^	269.0806	−0.88	254.0574, 237.0549, 213.0911	AR
				[M − H]^−^	267.0661	+0.89	252.0429, 223.0403, 195.0451	AR
130	11.13	Senkyunolide F	C_12_H_14_O_3_	[M − H]^−^	205.0867	+0.81	187.0772, 161.0971	AR
131	11.20	Butylphthalide	C_12_H_14_O_2_	[M + H]^+^	191.1066	−0.09	173.0959, 163.1118, 149.0597, 145.1012, 135.0441	AES
132	11.22	Senkyunolide K	C_12_H_16_O_3_	[M − H]^−^	207.1024	+0.88	163.1128, 159.0817	AES
133	11.39	Odoratin	C_17_H_14_O_6_	[M − H]^−^	313.0719	+1.21	298.0452, 193.0506, 134.0374	AR
134	11.61	6,7-Epoxyligustilide	C_12_H_14_O_3_	[M − H]^−^	205.0866	+0.69	161.0972, 106.0424	AR
135	11.69	Senkyunolide G	C_12_H_16_O_3_	[M − H]^−^	207.1026	+1.03	163.1021, 161.0972, 106.0124	AES
136	11.84	Coniferyl ferulate	C_20_H_20_O_6_	[M − H]^−^	355.1187	+1.07	311.1292, 267.1392, 223.1486, 189.0918, 167.0352, 123.0453	AR
137	12.19	Senkyunolide E or isomer	C_12_H_12_O_3_	[M + H]^+^	205.0859	−0.15	187.0753, 169.0634, 159.0806	AES
				[M − H]^−^	203.0711	+0.87	182.9877, 174.0323, 160.0166	AR
138	12.30	Soyasaponin I	C_48_H_78_O_18_	[M + H]^+^	943.5261	−0.03	599.3937, 441.3727, 423.3622	AR
				[M − H]^−^	941.5103	−0.19	795.1194, 615.3898	AR
139	12.74	Senkyunolide E or isomer	C_12_H_12_O_3_	[M − H]^−^	203.0712	+0.93	174.0321, 159.0814	AR
140	12.90	Agroastragaloside IV	C_49_H_80_O_20_	[M − H]^−^	987.5162	+0.25	-	AR
141	13.06	Astragaloside I	C_45_H_72_O_16_	[M + H]^+^	869.4891	−0.19	653.4056, 455.3528, 437.3419, 419.3309	AR
142	13.08	Senkyunolide A	C_12_H_16_O_2_	[M + H]^+^	193.1223	−0.19	175.1118, 147.1168, 137.0597, 105.0698	AES
143	13.18	Astroolesaponins A	C_48_H_76_O_18_	[M − H]^−^	939.4955	+0.69	921.4890, 613.3750, 455.3525	AR
144	13.32	9-Octadecenedioic acid	C_18_H_32_O_4_	[M − H]^−^	311.2226	+0.91	293.2125, 275.2008, 223.1705	AR
145	13.50	Isoastragaloside I	C_45_H_72_O_16_	[M + H]^+^	869.4888	−0.62	653.4056, 455.3528, 437.3419, 419.3309, 217.0707, 199.0603	AR
146	13.80	Neoastragaloside I	C_45_H_72_O_16_	[M + H]^+^	869.4896	+0.29	653.4056, 455.3528, 437.3419	AR
147	13.90	9,10-dihydroxy-12Zoctadecenoic acid	C_18_H_34_O_4_	[M − H]^−^	313.2383	+1.00	295.2278, 277.2173, 183.1390	AES, AR
148	13.91	13-Hydroxy-9,11-octadecadienoic acid	C_18_H_32_O_3_	[M + H]^+^	297.2424	−0.17	279.2318, 261.2215, 243.2112, 184.0993, 147.1168	AES, AR
				[M − H]^−^	295.2278	+0.98	277.2174, 195.1391	AR
149	14.05	10-Angeloylbutylphthalide	C_17_H_20_O_4_	[M + H]^+^	289.1434	−0.02	189.0910, 171.0804	AES
150	14.27	Capsidiol or isomer	C_15_H_24_O_2_	[M − H]^−^	235.1703	+1.01	214.9946, 191.0714, 163.0767, 145.9308	AR
151	14.53	Tokinolide C	C_24_H_28_O_4_	[M + H]^+^	381.2061	+0.22	335.2009, 191.1067, 173.0961	AES
152	14.78	Tokinolide B	C_24_H_28_O_4_	[M + H]^+^	381.2060	−0.04	335.2009, 191.1067, 173.0961	AES
153	14.79	Riligustilide	C_24_H_28_O_4_	[M + H]^+^	381.2060	−0.04	191.1066, 173.0961	AES
154	14.79	E, E′-3. 3′,8. 8′-isodiligustilide	C_24_H_26_O_4_	[M + H]^+^	379.1904	+0.04	361.1801, 191.1066	AES
155	14.80	Ligustilide	C_12_H_14_O_2_	[M + H]^+^	191.1066	−0.14	173.0961, 163.1118, 145.1012	AES
156	15.01	Levistolide A	C_24_H_28_O_4_	[M + H]^+^	381.2060	−0.12	191.1067, 173.0961	AES
157	15.06	Angelicide	C_24_H_28_O_4_	[M + H]^+^	381.2059	−0.44	365.9087,191.1067, 173.0961	AES
158	15.30	Linolenic acid	C_18_H_30_O_2_	[M − H]^−^	277.2173	+1.08	259.2053, 205.1598	AR
159	15.56	Quercetin	C_15_H_10_O_7_	[M − H]^−^	301.0356	+4.22	-	AR
160	15.57	Linoleic acid	C_18_H_32_O_2_	[M − H]^−^	279.2328	+0.96	261.2234, 227.1188	AES, AR
161	15.90	Palmitic acid	C_16_H_32_O_2_	[M − H]^−^	255.2329	+1.00	154.3097	AR

**Table 2:** EC_50_ values of eight active compounds from DBD for relaxing uterine smooth muscle precontracted with OT (50 ng/mL) or KCl (60 mM).

Components	EC_50_ (μM) (OT (50 ng/mL))	EC_50_ (μM) (KCl (60 mM))
Quercetin	35.49	8.911
Ligustilide	47.23	11.15
Calycosin	59.82	12.10
Ferulic acid	>160	19.80
Senkyunolide I	>160	81.52
Ononin	>160	87.73
Formononetin	>160	>160
Calycosin-7-O-beta-D-glucoside	>160	>160

EC_50_ values for compounds without clear concentration-dependent effects (5–160 μM) are rough estimates.

**Table 3:** Primer sequences.

Name	Forward Primer	Reverse Primer
*Il6*	CGGAGAGGAGACTTCACAGAGGA	TTTCCACGATTTCCCAGAGAACA
*Pgf2a*	GCCTTCTTGGGACTGATGCT	AGCCTCCGACTTGTGAAGTG
*Ptgfr*	CAAACACAACCTGCCAGACG	AGCAGAAACGATGCCTTGGA
*Tnfa*	CCCTCACACTCAGATCATCTTCT	GCTACGACGTGGGCTACAG
*Actb*	GGCTGTATTCCCCTCCATCG	CCAGTTGGTAACAATGCCATGT

## Data Availability

The original contributions presented in this study are included in the article and Supplementary Materials. Further inquiries can be directed to the corresponding authors.

## Supplementary Materials

The following supporting information can be downloaded at: https://www.mdpi.com/article/10.3390/ph19030520/s1, Figure S1. Typical tension recording profiles of isolated uterine muscle strips. Figure S2. Preliminary experimental data on indicators related to the dysmenorrhea model. Figure S3. Histological images of uterine tissue from primary dysmenorrhea female mice. Figure S4. PTGFR displayed strong binding affinity for all eight compounds (Calycosin, Calycosin-7-O-β-D-glucoside, Ferulic acid, Formononetin, Ligustilide, Ononin, Quercetin, Senkyunolide I). Table S1. Molecular docking results.

## Abbreviations

The following abbreviations are used in this manuscript:

DBD	Danggui Buxue Decoction
OT	Oxytocin
NSAIDs	Non-steroidal anti-inflammatory drugs
AES	Angelica sinensis
AR	Astragalus membranaceus
K-H	Krebs–Henseleit
IP_3_R	Inositol trisphosphate receptor
HE	Haematoxylin and eosin
PTGFR	Prostaglandin F receptor
IP_3_	Inositol 1,4,5-trisphosphate
PLC	Phospholipase C
