# Supplementary material for: Danggui Buxue Decoction and Its Active Constituents Inhibit Drug-Induced Uterine Contractions via L-Type Calcium Channels and the IP3/Ca2+ Pathway
Source: Pharmaceuticals (Basel). 2026 Mar 23;19(3):520. doi: 10.3390/ph19030520 (PMC13029447; doi:10.3390/ph19030520)
Supplement: Supplementary file 1 [file pharmaceuticals-19-00520-s001.zip › pharmaceuticals-4197527-supplementary.pdf]

Supplementary Material

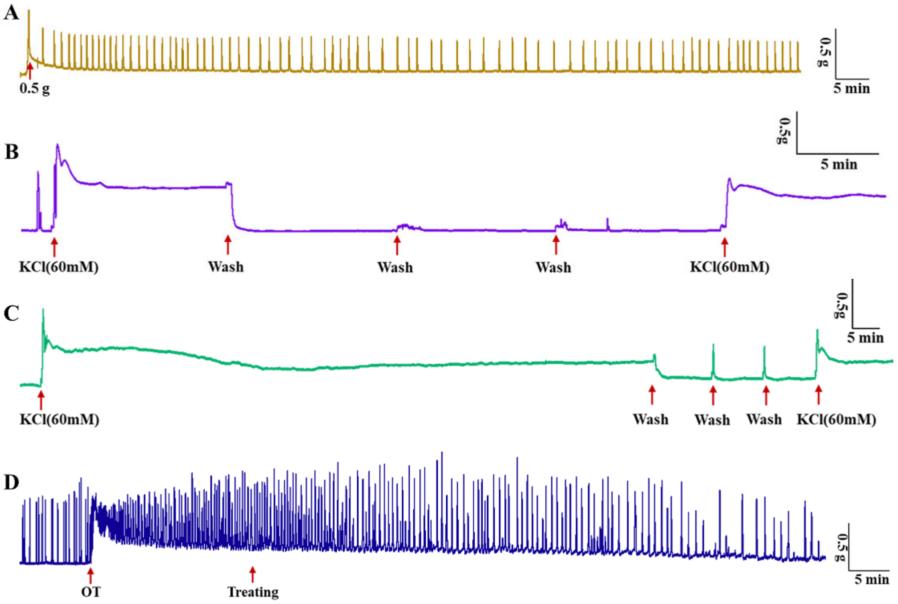

Figure S1. Typical tension recording profiles of isolated uterine muscle strips. (A) Schematic diagram of uterine muscle strip contraction after applying initial tension. (**B)** Verification of uterine smooth muscle strip activity. (**C)** Verification of uterine smooth muscle strip activity following drug administration. (**D)** Representative tracing of OT induced tension changes.

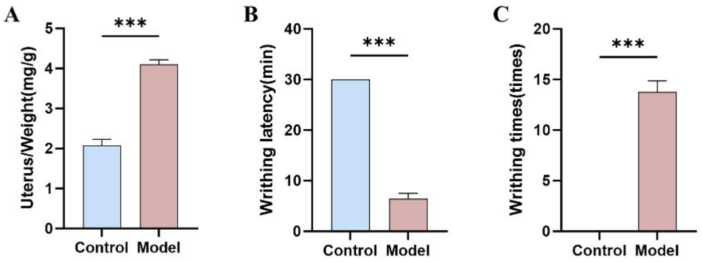

Figure S2. Preliminary experimental data on indicators related to the dysmenorrhea model. Uterine organ index (**A**), writhing latency (**B**), and writhing times (**C**) in primary dysmenorrhea model female mice during the pre-experimental phase. Data are presented as means ± SEM, n = 4. Compared with the model group, $^{***}P < 0.001$.

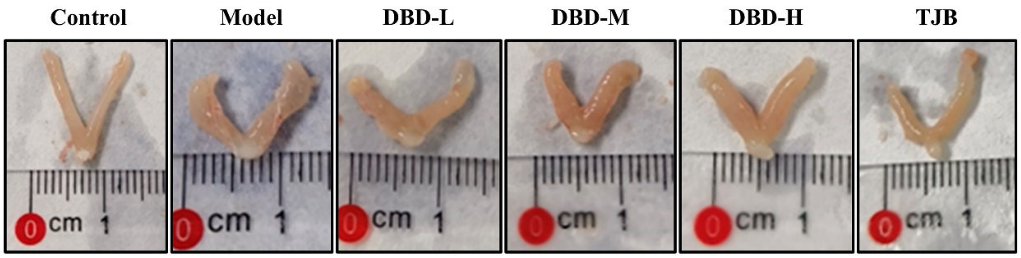

Figure S3. Histological images of uterine tissue from primary dysmenorrhea female mice.

Molecule Docking: The 3D structure of PTGFR (ID: 8IUK) was obtained from the PDB database, and the structures of the compounds were retrieved from PubChem and ChemSpider databases. Using PyMOL, water molecules and native ligands were removed from the protein. Hydrogen atoms were added using AutoDock, and subsequently, both the protein and compound ligands were processed with AutoDockTools and saved in PDBQT format. Molecular docking was performed using AutoDock, and the results were visualized with PyMOL. In this study, binding energies below –5.0 kcal/mol were considered indicative of strong ligand-receptor binding affinity.

Table S1. Molecular docking results for eight compounds with PTGFR.

Ligand	Binding Energy (kcal/mol)
Quercetin	-8.4
Ligustilide	-8.2
Calycosin-7-O-beta-D-glucoside	-8.1
Calycosin	-7.9
Ononin	-6.6
Senkyunolide I	-6.2
Ferulic acid	-6.2
Formononetin	-6.1

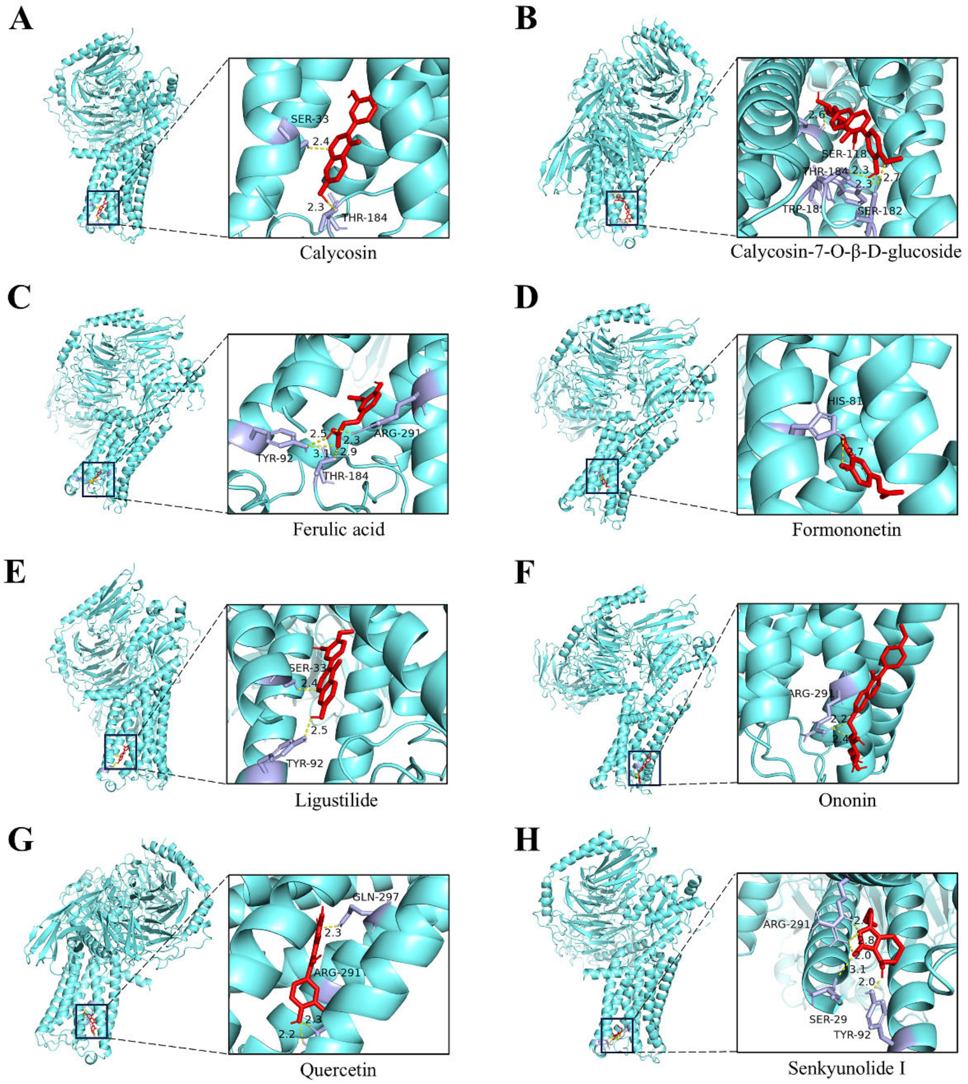

Figure S4. PTGFR displayed strong binding affinity for all eight compounds (Calycosin, Calycosin-7-O-β-D-glucoside, Ferulic acid, Formononetin, Ligustilide, Ononin, Quercetin, Senkyunolide I).
